# Dysferlin Forms a Dimer Mediated by the C2 Domains and the Transmembrane Domain In Vitro and in Living Cells

**DOI:** 10.1371/journal.pone.0027884

**Published:** 2011-11-14

**Authors:** Li Xu, Sandeep Pallikkuth, Zhanjia Hou, Gregory A. Mignery, Seth L. Robia, Renzhi Han

**Affiliations:** Department of Cell and Molecular Physiology, Stritch School of Medicine, Loyola University Medical Center, Maywood, Illinois, United States of America; German Cancer Research Center, Germany

## Abstract

Dysferlin was previously identified as a key player in muscle membrane repair and its deficiency leads to the development of muscular dystrophy and cardiomyopathy. However, little is known about the oligomerization of this protein in the plasma membrane. Here we report for the first time that dysferlin forms a dimer in vitro and in living adult skeletal muscle fibers isolated from mice. Endogenous dysferlin from rabbit skeletal muscle exists primarily as a ∼460 kDa species in detergent-solubilized muscle homogenate, as shown by sucrose gradient fractionation, gel filtration and cross-linking assays. Fluorescent protein (YFP) labeled human dysferlin forms a dimer in vitro, as demonstrated by fluorescence correlation spectroscopy (FCS) and photon counting histogram (PCH) analyses. Dysferlin also dimerizes in living cells, as probed by fluorescence resonance energy transfer (FRET). Domain mapping FRET experiments showed that dysferlin dimerization is mediated by its transmembrane domain and by multiple C2 domains. However, C2A did not significantly contribute to dimerization; notably, this is the only C2 domain in dysferlin known to engage in a Ca-dependent interaction with cell membranes. Taken together, the data suggest that Ca-insensitive C2 domains mediate high affinity self-association of dysferlin in a parallel homodimer, leaving the Ca-sensitive C2A domain free to interact with membranes.

## Introduction

The functionality of muscle cells is dependent upon the integrity of the plasma membrane (sarcolemma). A membrane repair mechanism involving multiple proteins such as dysferlin [1], [2], [3], calpain [4], annexins A1/A2/A5 [3], [5], [6] and MG53 [7], [8] has been identified to restore the sarcolemmal integrity upon membrane damage. Defects in the membrane repair machinery are detrimental to normal muscle function and health. For example, genetic defects in the *DYSF* gene lead to the development of multiple muscular dystrophies. Such dysferlinopathies include limb-girdle muscular dystrophy type2B (LGMD2B) [9], [10], Miyoshi myopathy [10] and a distal anterior compartment myopathy [11]. In addition, dysferlin deficiency also causes the development of cardiomyopathy [2], [12], [13], [14].

Dysferlin is expressed in tissues including skeletal muscle, heart, kidney, placenta, lung, and brain [9]. Despite the progress in establishing the function of dysferlin in muscle membrane repair [1], [2], [3], [15], little is known about how dysferlin exerts its function. Dysferlin is a 230 kDa type II transmembrane protein, belonging to the ferlin-1-like protein family [9], [16]. All ferlin-1-like proteins contain multiple C2 domains that were known to possess the characteristics of Ca^2+^-dependent phospholipid binding activities [17], [18]. Indeed, the first C2 domain (designated as C2A) of dysferlin was observed to bind to phospholipids in a Ca^2+^ dependent fashion [17], [18]. Mutations within the C2A domain of dysferlin reduced the Ca^2+^-facilitated phospholipid binding activity [17], [18]. However, the other C2 domains in dysferlin exhibited weaker Ca^2+^-independent or no binding to phospholipids [18]. This raises an interesting yet unresolved question: what is the role of the other six C2 domains? Previous studies demonstrated that C2 domains, in addition to mediating Ca^2+^-sensitive membrane binding activity, could also mediate protein-protein interactions [19], [20], [21]. In particular, the C2 domains dimerization has been reported for RIM1α [22] and Munc13 [23]. To test whether the C2 domains in dysferlin mediate dysferlin oligomerization, we employed a combination of biochemical and optical approaches to study the self-interaction of dysferlin *in vitro* and in living cells.

## Results

### Endogenous dysferlin is present as a high molecular mass species in vitro

**Figure 1:**
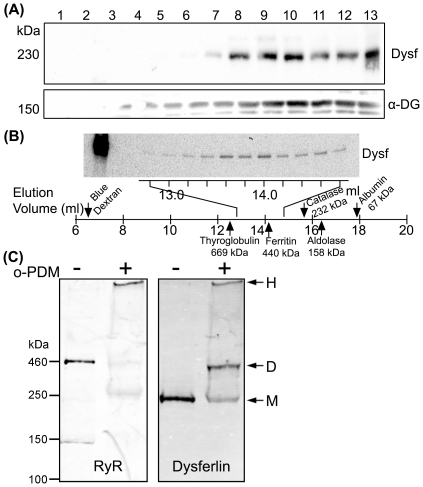
Biochemical analyses of endogenous dysferlin from skeletal muscle in vitro. (A) Dysferlin-enriched fraction from DEAE column (150 mM NaCl) was run onto a 5–30% linear sucrose gradient. Total 13 fractions were taken from the top to the bottom of the gradient and analyzed by SDS-PAGE. Dysferlin (Dysf) was detected in the heavy fractions overlapping the dystrophin-glycoprotein complex detected by the α-dystroglycan (DG) antibody. (B) Gel filtration profile of purified dysferlin. The elution volumes (ml) were shown below the lanes. The first lane was the starting material - purified dysferlin. The standards elution profiles (marked with the arrows: thyroglobulin, 669 kDa; ferritin, 440 kDa; catalase, 232 kDa; aldolase, 158 kDa, albumin, 67 kDa) off the column were linear and had an R^2^ value of 0.974. (C) Rabbit skeletal muscle microsomes were treated with or without 100 M o-PDM and the samples were analyzed by SDS-PAGE and western blotting analysis. Dysferlin was detected at 230 kDa and 460 kDa (using ryanodine receptor (RyR) as a molecular marker).

We used ion exchange chromatography to enrich dysferlin from rabbit skeletal muscle microsomes. When digitonin (1%)-solubilized KCl-washed microsomes of rabbit skeletal muscle was applied to DEAE cellulose and step-eluted with increasing NaCl concentrations, the 150 mM NaCl wash fraction was enriched with dysferlin. We then ran the dysferlin-enriched fraction from the DEAE column onto a linear sucrose gradient (5–30%) and probed the fractions with a dysferlin antibody (Hamlet-1). As shown in **Fig. 1A**, dysferlin migrated into heavier fractions 8–13, suggesting that dysferlin exists as high-molecular-weight species (through self-association or binding to some other proteins).

To obtain further information about the sizes of dysferlin complexes, we analyzed purified dysferlin (in 1% CHAPS) by size exclusion FPLC over Superose 6 columns. The elution profile was shown in **Fig. 1B**. Purified dysferlin was detected with the peak in the fractions near ferritin (440 kDa), twice of the apparent MW of monomeric dysferlin (230 kDa). This result indicates that dysferlin forms a dimer in solution.

To further investigate the high MW species, we incubated rabbit skeletal muscle microscomes with 100 µM bifunctional maleimide cross-linker N,N′-o-phenylenedimaleimide (o-PDM; rigid 6 Å), and then analyzed the samples by SDS-PAGE. In o-PDM treated samples dysferlin existed as two discrete bands, one at 230 kDa and the other at 460 kDa (**Fig. 1C**). These data consistently support that dysferlin *in vitro* forms a homodimeric complex.

### Dysferlin forms a dimer in living HEK293 cells

**Figure 2:**
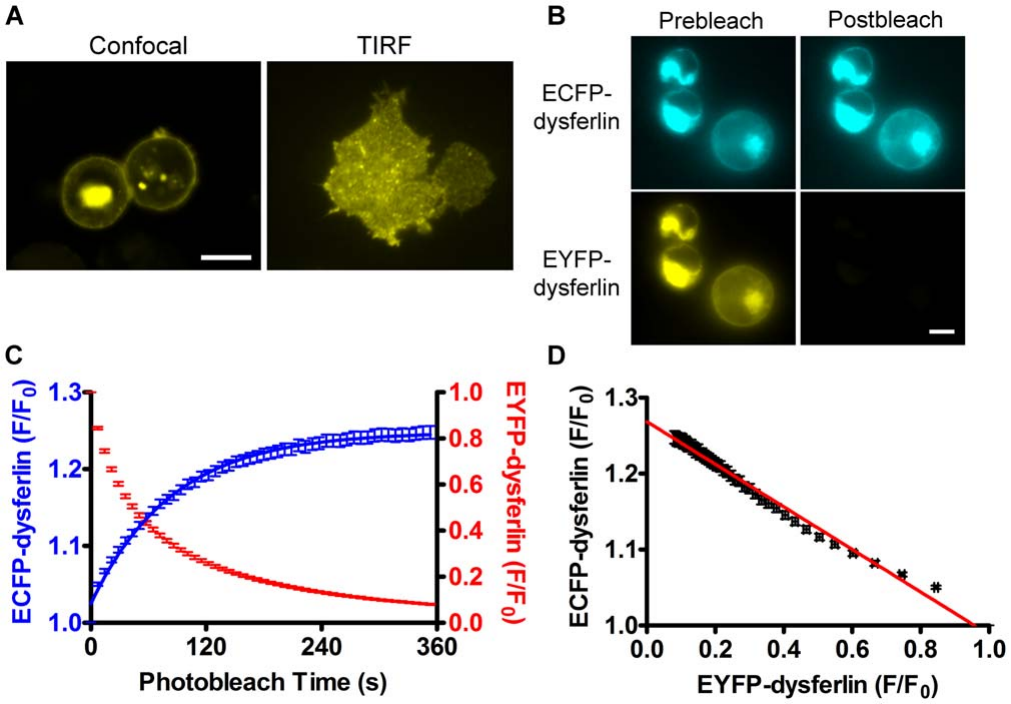
Dysferlin self-interaction in living HEK293 cells shown by acceptor-selective photobleaching FRET assay. (A) Confocal microscopy and total internal reflection fluorescence (TIRF) microscopy images of YFP-dysferlin expressed in HEK293 cells. Scale bars: 10 µm. (B) CFP-dysferlin and YFP-dysferlin fluorescence images before (Prebleach) and after (Postbleach) YFP-selective photobleaching. Scale bar: 5 µm. (C) Quantitative analysis of CFP-dysferlin and YFP-dysferlin fluorescence intensities (F/F_0_) during YFP-selective photobleaching. (D) The relationship between the normalized fluorescence of CFP-dysferlin and the normalized fluorescence of YFP-dysferlin during progressive photobleaching was linear, consistent with a homodimeric dysferlin complex.

To test whether dysferlin forms self-associated complex in living cells, we performed the FRET assay on transiently transfected HEK293 cells (Stratagene). We have previously used this assay to investigate the interactions of muscle proteins including SERCA and phospholamban (PLB) [24], [25], [26]. HEK293 cells do not express dysferlin endogenously, allowing us to study the dysferlin self-interaction without interference from the endogenous dysferlin. Confocal microscopy and total internal reflection fluorescence (TIRF) microscopy showed that CFP/YFP-dysferlin was localized at the cell membrane and in intracellular vesicles in HEK293 cells (**Fig. 2A**).

Energy transfer between CFP-dysferlin and YFP-dysferlin was detected by acceptor-selective photobleaching. Selective photobleaching of YFP resulted in a concomitant increase in CFP-dysferlin fluorescence (**Fig. 2B,C**), indicating FRET from CFP-dysferlin to YFP-dysferlin. To probe the stoichiometry of the oligomeric complex that gives rise to FRET, the relationship of CFP and YFP fluorescence emission was examined as described previously [24], [27]. A highly linear relationship was observed between CFP-dysferlin and YFP-dysferlin emissions during the progressive YFP photobleaching (**Fig. 2D**), consistent with a homodimeric complex. This is in contrast to the highly nonlinear donor-acceptor relationships previously observed for PLB [24] and phospholemman [25] homooligomers with the subunit number >2 [27]. The linear regression *y*-intercept (**Fig. 2D**) also provides a measure of the FRET_max_ between CFP-dysferlin and YFP-dysferlin (26.8±0.1%, N = 187). This FRET value is significantly higher than the nonspecific FRET for membrane proteins that was characterized previously in the same setup (∼4%) [28], affirming the specificity of dysferlin self-interaction.

Dysferlin vesicles were highly mobile in HEK293 cells. These vesicles, when moving into or out of the focus, may interfere with the fluorescence measurement during progressive YFP photobleaching which usually takes around 6 minutes, thus affecting the FRET measurement. As a complementary approach, we also measured FRET using the “three-cube” fluorescence microscopy [29], [30], [31] as this method is fast and nondestructive. Since the movement of the cells and the vesicles during fast three-cube imaging is negligible, FRET calculation can be performed in a pixel-by-pixel fashion after background subtraction. The calculated FRET efficiency averaged from 3210 pixels was 20.6±2.1%, consistent with the acceptor-selective photobleaching method.

### The C2 domains and the transmembrane domain mediate dysferlin dimerization

**Figure 3:**
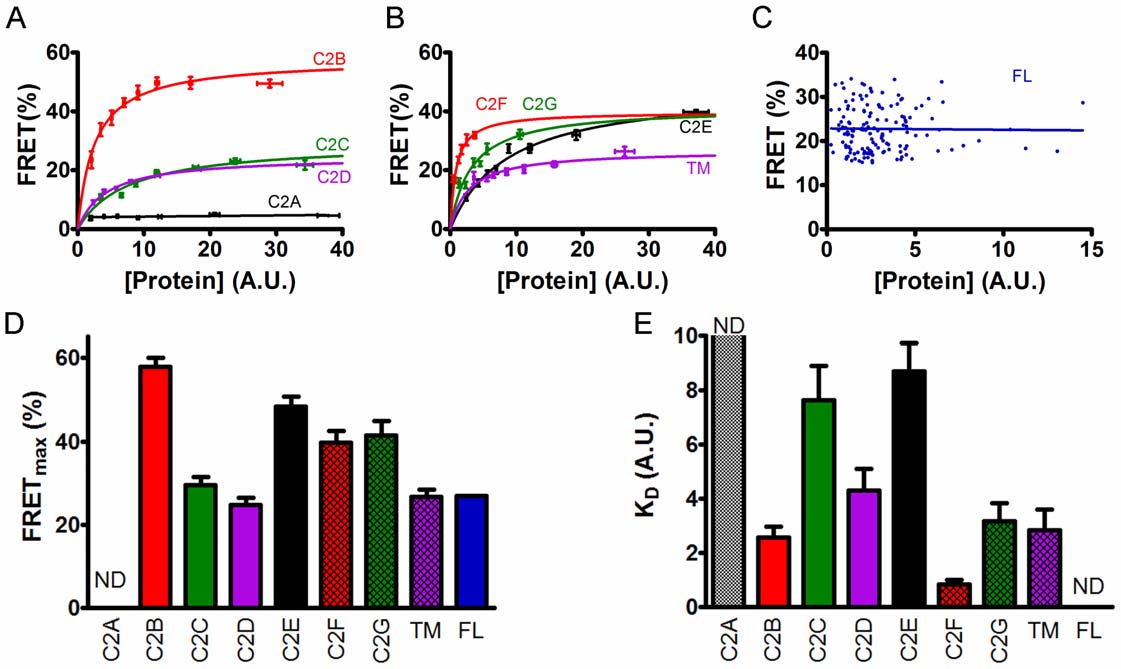
Mapping the determinants of dysferlin dimerization. (A) Dependence of FRET efficiencies on the protein expression levels of C2A to C2D domains of dysferlin. Hyperbolic fitting showed that the C2B (red), C2C (green) and C2D (purple) domains of dysferlin all mediate the self-interaction. There was no FRET for C2A construct (black). (B) Dependence of FRET efficiencies on the protein expression levels of C2E to C2G domains and the transmembrane (TM) domain of dysferlin. Hyperbolic fitting showed that the C2E (black), C2F (red), C2G (green) and TM (purple) domains of dysferlin also mediate the self-interaction. In A and B, the data were pooled for easy comparison. (C) Independence of dysferlin dimer FRET efficiencies on acceptor concentrations within the range examined. (D) Summary of FRET_max_ values obtained by fitting (expressed as mean ± S.E.M.). (E) Summary of *K_D_* values obtained by fitting (expressed as mean ± S.E.M.). ND: not determined.

Dysferlin contains multiple C2 domains, which are potentially involved in mediating dysferlin dimerization. To test this, we generated the CFP and YFP fusion constructs containing each individual C2 domain, and examined their self-interactions in HEK293 cells using FRET assays. Interestingly, the C2A domain did not bind to itself while all the other C2 domains showed significant self-interactions (**Fig. 3A&B**). To obtain the relative binding energetics, we surveyed large populations of cells expressing fluorescently tagged proteins and compared each cell's FRET efficiency with its YFP fluorescence intensity, which is an index of protein expression [24], [25], [28], [32], [33]. For C2B to C2G, FRET efficiency increased with increasing protein expression - a relationship that can be approximated by a hyperbolic fit in the form *y* = (FRET_max_)*x*/(*K_D_*+*x*). The parameter FRET_max_ is the maximal FRET, taken to represent the intrinsic FRET of the bound complex, and *K_D_* represents the protein concentration at which half-maximal FRET is achieved [dissociation constant, in arbitrary units (AU)]. These data are pooled and averaged in **Fig. 3A&B** to aid in the comparison of the FRET concentration dependences. The mean FRET_max_ and *K_D_* values are summarized in **Fig. 3D** and **Fig. 3E**, respectively. Beside the C2 domains, we observed that the transmembrane domain of dysferlin is also involved in the dimerization (**Fig. 3B**). We also tried to measure the FRET_max_ and *K_D_* of full-length dysferlin using the same method. However, we observed no significant changes in measured FRET efficiency with a broad range of acceptor protein concentrations (from 0.23 to 15 in A.U.) (**Fig. 3C**), indicating that *K_D_* for dysferlin is very small, at least smaller than the lowest concentration examined (0.23 A.U.).

### Dysferlin forms self-associated complex in living adult skeletal muscle fibers

**Figure 4:**
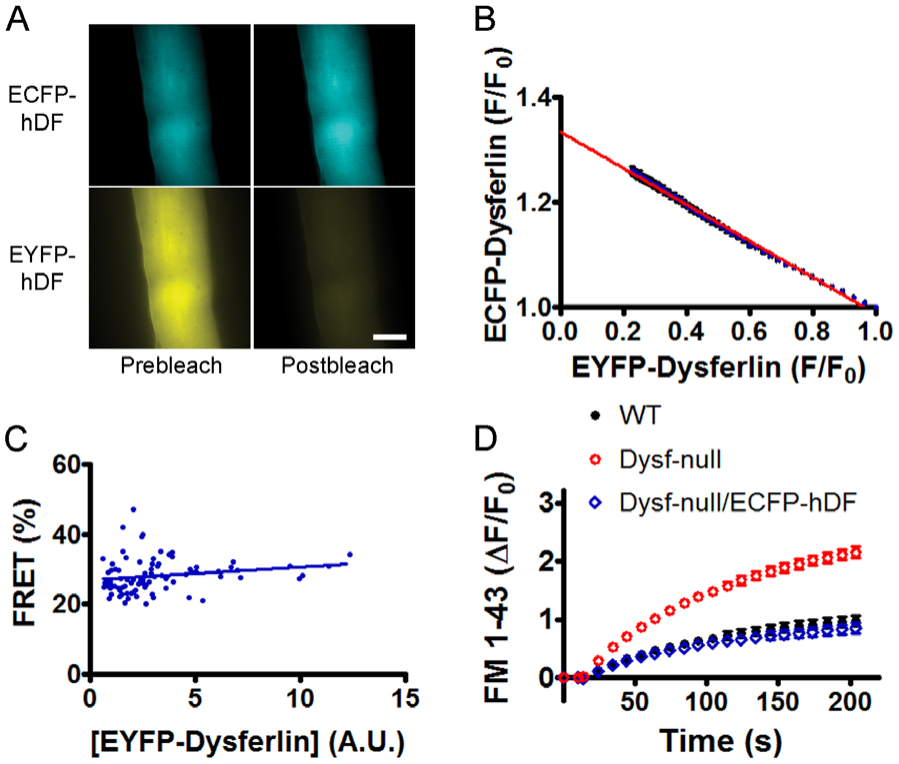
FRET analyses of adult FDB muscle fibers transfected with CFP-dysferlin and YFP-dysferlin. (A) CFP-dysferlin and YFP-dysferlin fluorescence images before (Prebleach) and after (Postbleach) YFP-selective photobleaching. Scale bar: 20 µm. (B) Linear relationship between CFP-dysferlin and YFP-dysferlin fluorescence suggests dysferlin forms a dimer in FDB muscle fibers. (C) Independence of dysferlin dimer FRET efficiencies on YFP-dysferlin concentrations within the range examined. (D) Membrane repair defect in dysferlin-deficient muscle fibers was corrected by the CFP-dysferlin, suggesting that the fluorescent protein tag on dysferlin does not compromise the function of dysferlin.

To test whether dysferlin also forms dimers in living muscle fibers, *flexor digitorum brevis* (FDB) muscles of *dysferlin*-deficient mice were transfected with CFP-dysferlin and YFP-dysferlin by electroporation [34], [35], and individual FDB muscle fibers were isolated by collagenase digestion [36]. The positively transfected FDB muscle fibers (**Fig. 4A**) were similarly assayed by acceptor-selective photobleaching method and three-cube fluorescence microscopy. As in HEK293 cells, selective YFP photobleaching resulted in a concomitant increase in CFP-dysferlin fluorescence in FDB muscle fibers (**Fig. 4B**). The inverse relationship between CFP-dysferlin and YFP-dysferlin fluorescence intensity during YFP-dysferlin photobleaching was also highly linear (**Fig. 4C**), indicating that dysferlin in adult skeletal muscle fibers also forms a dimer. **Fig. 4D** showed no significant changes in measured FRET efficiency over all achievable protein concentrations (from 0.23 to 30 in A.U.), again suggesting that the *K_D_* for dysferlin dimer is too low to be measured. The FRET_max_ (33.3±0.1%, taken from the linear regression *y*-intercept in **Fig. 4C**) measured from FDB muscle fibers were higher than that in HEK293 cells, suggesting that muscle cell environment favors a tighter dysferlin dimer formation.

Finally, to ascertain that the fluorescent protein tag does not impact the function of dysferlin, we examined the membrane repair capacity of the muscle fibers with or without CFP-dysferlin. When FM 1–43-labeled sarcolemma were irradiated with an 880-nm infra-red laser beam at full power, the FM 1–43 fluorescence intensities were increased at the irradiation site indicating of membrane damage (**Fig. 4E**). In contrast to wild-type muscle fibers, the dysferlin-deficient muscle fibers lost the ability to stop the fluorescence increase (**Fig. 4E**) due to compromised membrane repair as reported previously [1], [2]. However, CFP-dysferlin, when introduced into dysferlin-deficient muscle fibers, fully restored the membrane repair capacity in these cells (**Fig. 4E**). These data suggest that the fluorescent protein tag does not impair the structure and/or function of dysferlin when fused in the N terminus of dysferlin.

### Fluorescence correlation spectroscopy and photon counting histogram analyses

**Figure 5:**
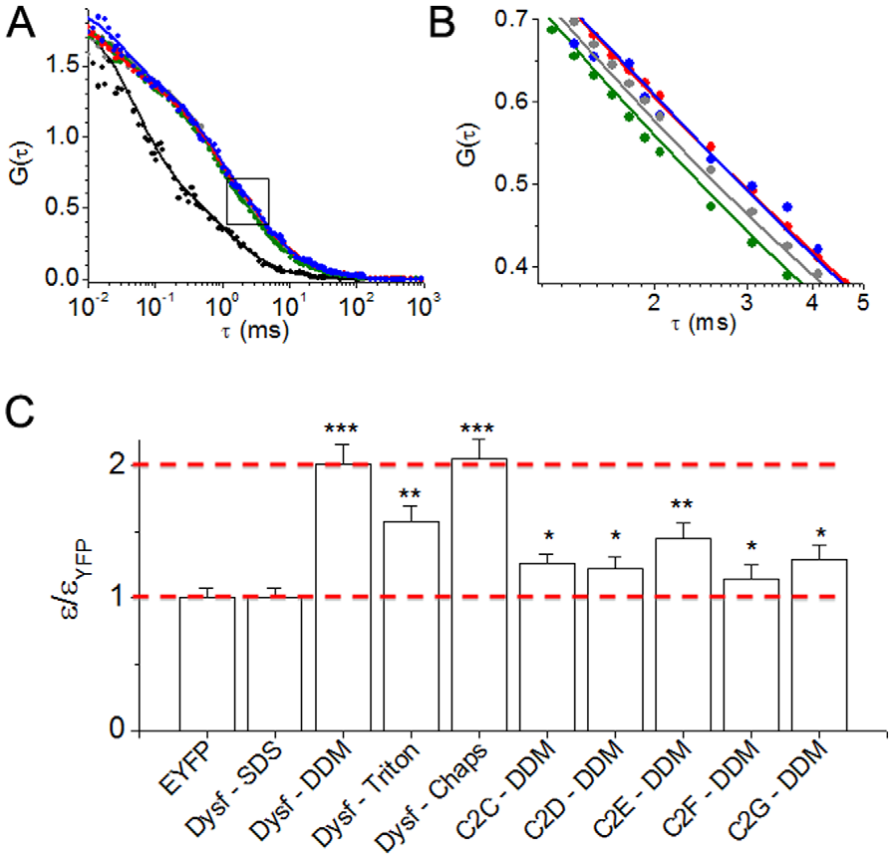
Fluorescence correlation spectroscopy and photon counting histogram analyses of dysferlin dimerization. (A) FCS data obtained from YFP control (black) showed a significantly faster diffusion compared to full length YFP dysferlin in detergent solution. The boxed region is enlarged and reproduced in the next panel. (B) Diffusion of YFP-dysferlin in 1% CHAPS (red) and 0.5% DDM (blue) were well described by a single species diffusion model (**Equation 1**), with a correlation time of 2.7 ms. This the apparent diffusion time constant was decreased in SDS (green) to 2.1 ms, suggesting a smaller species half the size of YFP-dysferlin in CHAPS or DDM. Triton X-100 solubilization yielded a YFP-dysferlin correlation (gray) that was intermediate between SDS and DDM/CHAPS. These data were best described by a fit to 2 species differing in molecular weight by 2-fold (**Equation 3**). The data suggest that the dysferlin complex is partially destabilized by Triton X-100, but some dimers remain. (C) PCH analysis yielded the molecular brightness (ε) of diffusing species, normalized to the brightness of control monomeric YFP. The molecular brightness of monomers and dimers are highlighted (dotted red lines). YFP-dysferlin in CHAPS and DDM had a molecular brightness exactly twice the measured brightness of monomeric YFP, suggesting stable dimers in these detergents. YFP-dysferlin in Triton X-100 and individual C2-domains all yielded intermediate molecular brightness, which is compatible with a mixture of monomers and dimers. *** indicates p<0.001, ** p<0.01 and * p<0.05 when compared to the molecular brightness of YFP.

To further investigate dimerization of dysferlin in *in vitro*, we performed fluorescence correlation spectroscopy on enhanced YFP-labeled dysferlin or free YFP diffusing in detergent solution. As expected, there was a marked difference in the correlation time of the small, freely diffusible YFP compared to YFP fused to dysferlin in detergent micelles. This is evident as a significantly right-shifted correlation of YFP-dysferlin compared to YFP (**Fig. 5A**, black). Interestingly, we also observed differential diffusion of YFP-dysferlin in various detergents (**Fig. 5B**). The fluorescence correlation of YFP-dysferlin in two mild non-charged detergents (1% CHAPS, **Fig. 5B**, red and 0.5% DDM, **Fig. 5B**, blue) were well described by a single species diffusion model (**Equation 1**), with a correlation time of 2.7 ms. This corresponds to a diffusion coefficient (D) of 33 µm^2^ s^−1^ (**Table 1**). However, in a negatively-charged denaturing detergent SDS (**Fig. 5B**, green) YFP-dysferlin correlation time was decreased to 2.1 ms (D = 42 µm^2^ s^−1^), suggesting a smaller molecular weight species. Using a model of diffusion of a spherical shape (**Equation 3**), FCS measurements yielded estimates of hydrodynamic radii of 59 and 75 Å for dysferlin in SDS and DDM/Chaps, corresponding to apparent molecular weights of 315 and 649 kD, respectively. Note that these apparent molecular weights are probably overestimates, since dysferlin is not expected to have a spherical shape. The two-fold difference in the apparent molecular weights is suggestive of a DDM/Chaps-stable dimer that is dissociated by SDS. Triton X-100 solubilization yielded a YFP-dysferlin correlation (**Fig. 5B**, gray) that was intermediate between SDS and DDM/Chaps, consistent with a mixture of monomers and dimers. Indeed, a 2-species fit (**Equation 2**) revealed that 83% of the molecules diffused with a correlation time of 2.1 ms, and 17% diffused with a correlation time of 2.7 ms (**Table 1**). The data suggest that the dysferlin complex is partially destabilized by Triton X-100, but some dimers remain.

**Table 1:** Summary of FCS experiments.

Sample	τ_D_ (ms)	D (µm^2^ s^−1^)	r_h_ (Å)
EYFP	0.93±0.05	0.95	26.1±1.9
Dysferlin in SDS	2.12±0.11	0.42±0.03	59.2±4.2
Dysferlin in CHAPS	2.69±0.13	0.33±0.02	75.3±5.3
Dysferlin in DDM	2.72±0.14	0.33±0.02	75.9±5.4
Dysferlin in Triton	2.12±0.112.72±0.14	0.42±0.030.33±0.02	59.4±4.276.1±5.4

Parameters shown are observed diffusion correlation time (τ_D_), diffusion coefficient (D), and apparent hydrodynamic radius (r_h_).

We also performed PCH analysis of detergent solubilized YFP-dysferlin to determine the molecular brightness (ε) of diffusing species. Histograms were fit with a two species model [37] in which one component was background autofluorescence. For YFP and YFP-dysferlin in SDS, the remaining component gave a molecular brightness of 4900 cpsm, which compares well with previous measurements of monomeric fluorescent proteins using low excitation power [38], [39]. YFP-dysferlin in CHAPS and DDM had a molecular brightness exactly twice the measured brightness of monomeric YFP (**Fig. 5C**), suggesting stable dimers in these detergents. The photon counting statistics were not sufficient for a 3-species fit of data obtained from YFP-dysferlin in Triton X-100, but this detergent yielded an apparent intermediate molecular brightness (**Fig. 5C**), which is compatible with a mixture of monomers and dimers. Similar results were obtained for individual C2 domains. **Fig. 5C** shows the results of PCH analysis of C2C-C2G, which yielded intermediate molecular brightness values consistent with mixtures of monomer and dimer species. These data are consistent with FRET experiments that showed that individual C2 domains were capable of dimerization, but with reduced affinity compared to full-length dysferlin (**Fig. 4**).

## Discussion

In the present work, we examined the stoichiometry of dysferlin using biophysical and biochemical approaches, and demonstrated for the first time that dysferlin forms a homodimer through the transmembrane domain and C2 domains.

Both sucrose gradient fractionation and gel filtration experiments showed that dysferlin existed as high MW species. SDS-PAGE analysis of cross-linked dysferlin revealed an apparent MW of 460 kDa, twice that of monomeric dysferlin, suggesting that dysferlin dimerizes *in vitro*. Dysferlin dimerization *in vitro* is further supported by PCH experiments that showed YFP-dysferlin in mild detergent (DDM) had a two-fold higher molecular brightness vs. YFP alone. PCH and FCS experiments also showed that the dimers were partially dissociated by triton X-100, and fully dissociated by SDS, which is compatible with a model of specific avid self-interactions of YFP-dysferlin.

Dysferlin dimerization is also suggested by FRET experiments in live cells. Using acceptor-photobleaching FRET and E-FRET, we consistently observed significant energy transfer between ECFP-dysferlin and EYFP-dysferlin in both HEK293 cells and muscle fibers. The FRET efficiency depends on the donor-to-acceptor separation distance R according to the relationship described by Förster R = (R_0_)[(1/FRET_max_)−1)]^1/6^ [40], where R_0_ is the Förster radius (49.2 Å for the CFP-YFP pair [41]). The FRET_max_ values (26.8±0.1% and 33.3±0.1% in HEK293 cells and muscle fibers, respectively) obtained for dysferlin after the subtraction of 4% nonspecific FRET as previously described [24], [28] correspond to CFP-YFP probe separation distance (R) of 58.2 and 57.0 Å in HEK293 cells and muscle fibers, respectively. It is noteworthy that the probe separation distance measured in HEK293 cells was longer than that obtained from muscle fibers. Possibly dysferlin favors a more compact dimeric structure in skeletal muscle compared to heterologous cells.

The observed self-interactions mediated by many C2 domains and the transmembrane domain of dysferlin suggest that multiple regions of the dysferlin structure contribute to binding. The binding energies for individual domains are expected to be additive for the full-length protein, with exponential consequences for the dissociation constant, according to the relationship ΔG = −RT*ln(*K_D_*). For example, a doubling of binding energy would reduce the *K_D_* by more than 7-fold. Thus, the avid self-interaction of discrete C2 domain fragments accounts for the observation that an ensemble of many C2 domains in full length dysferlin yielded a *K_D_* that was too low to measure. The high binding affinity makes it likely that dysferlin subunits come together *en route* to the plasma membrane, and that dysferlin exists as an obligate dimer *in vivo*.

**Figure 6:**
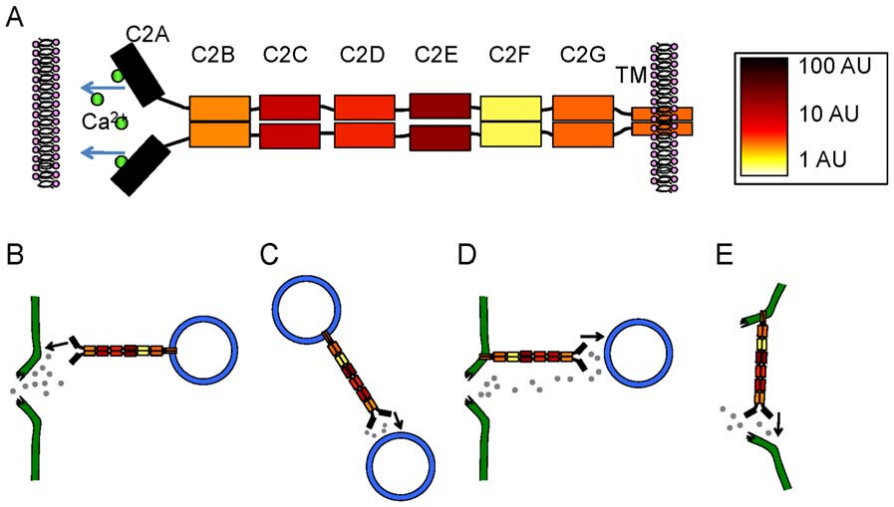
Schematic model of dysferlin protein. (A) We propose dysferlin forms a parallel homodimer through physical interactions of domains C2B to C2G and the transmembrane domains. The domains are color-coded according to measured dissociation constants to indicate the relative contribution of each domain to dimerization. After membrane damage, Ca^2+^ enters the cell and binds to the C2A domains. Ca^2+^-dependent C2A-membrane interactions result in bridging of two membranes: vesicle to plasma membrane (B, D), vesicles to vesicles (C), and two sides of the broken plasma membrane (E), thus promoting the formation of a dysferlin-membrane barrier surrounding membrane pores to accomplish membrane repair.

The present observations are summarized in a schematic diagram in **Fig. 6A**. In dysferlin, only the first C2 domain was reported to possess the Ca^2+^-facilitated phospholipid binding activity [17], [18] that is characteristic of C2 domains [42]. The present study showed that the rest of the C2 domains bind with differential affinity to their homologous partners to mediate dysferlin self-interactions. Based upon these observations, we believe that dysferlin dimer takes a parallel configuration (**Fig. 6A**). This arrangement is also consistent with FRET experiments, since an anti-parallel arrangement of full-length dysferlin would probably not give rise to FRET (though it would still be detected as a dimer by FCS, PCH, and other methods). The domains in Fig. 6A are color-coded to indicate their relative binding affinities. C2F contributes the most to binding, followed by the C2B, C2G, transmembrane domain, and then C2D, C2C and C2E. Since the C-terminus of dysferlin is anchored into a membrane, this parallel dimeric structure leaves the other end of dysferlin dimer with two C2A domains that do not bind to each other, but are free to interact with membranes (**Fig. 6A**). We propose that after membrane disruption, the high concentrations of Ca^2+^ flood into the cell, triggering membrane binding of the C2A domains. We envision several possible non-exclusive mechanisms of membrane repair mediated by dysferlin dimer localized at the sarcolemma and the vesicles (**Fig. 6B–E**). Ca^2+^ may trigger dysferlin anchored in intracellular vesicles to bind to the broken sarcolemma (**Fig. 6B**) or to other nearby vesicles (**Fig. 6C**). Dysferlin anchored in the sarcolemma could bind via C2A domains to intracellular vesicles (**Fig. 6D**) or to adjacent broken sarcolemma (**Fig. 6E**). In all cases, the vesicles (or membranes) are pulled together at the site of damage, accumulating a membrane barrier around the damage site and separating the high Ca^2+^ zone from the bulk cytoplasm. The previous observation that dysferlin-containing membrane patches are accumulated at the damaged muscle fibers [1] is consistent with this model.

Taken together, our data suggest that dysferlin possesses two types of C2 domains. C2A domains do not interact with another, but they mediate Ca^2+^-dependent phospholipid binding; C2B-F domains mediate protein-protein interactions, and together with the transmembrane domain they support dimerization of dysferlin. Understanding the dimeric nature of dysferlin is an essential step in revealing the mechanisms of dysferlin-mediated membrane repair and developing therapeutic drugs that could be useful in the treatment of dysferlinopathies.

## Materials and Methods

### Ethics Statement

All animal studies were reviewed and approved by the Institutional Animal Care and Use Committee of Loyola University Chicago (LU#202288 and LU#202769). Mice were maintained at Loyola University Medical Center Animal Facility in accordance with animal usage guidelines.

### Biochemical analysis of skeletal muscle

KCl-washed membrane preparations were prepared from rabbit skeletal muscles as previously described [43], and solubilized with 1% digitonin. Digitonin (1%)-solubilized membrane proteins were diluted 10 fold, applied to a 1-ml DEAE-cellulose column (DE-52, Whatman) and sequentially eluted with increased NaCl concentrations (mM): 50, 100, 150, 200, 300, 400, 500 and 750. The elution fractions were examined by SDS-PAGE and western blotting analysis. Dysferlin-enriched fraction was loaded onto 10–30% sucrose gradients (Biocomp Instruments) and sedimented at 220,000× g for 2 h. 13 fractions were collected from the top (light) to the bottom (heavy) of the gradients, and examined by SDS-PAGE and western blotting analysis.

### Superose 6 FPLC

FPLC analyses were performed with an Amersham Biosciences AB Superose 6 HR 10/30 column. The standards elution profile off the column were linear and had an R^2^ value of 0.974. His6-tagged dysferlin was purified using His-tag Dynabeads (Invitrogen) from the HEK 293 cells transfected with Fugene 6 (Roche). The mobile phase was 1% CHAPS, 150 mm NaCl, 50 mm Tris (pH 7.5) and run at 0.4 ml/min. The column eluates were fractionated into 100-µl aliquots and resolved by 4–15% SDS-PAGE followed by Western blotting with a monoclonal anti-dysferlin antibody Hamlet (Novacastra).

### Crosslinking assays

Rabbit skeletal muscle microsomes were treated with or without 100 µM o-PDM (Sigma-Aldrich) on ice for 30 min, or 5 mM diamide for 10 min at room temperature. The reactions were stopped after by addition of 5 mM N-ethylmaleimide, followed by addition of Laemmli sample buffer. The samples were analyzed by SDS-PAGE and western blotting.

### Western blotting

Protein samples were separated by SDS-PAGE and transferred onto PVDF membranes. The mouse monoclonal antibodies anti-dysferlin (Hamlet, Novacastra), anti-ryanodine receptor (XA7 B6, Developmental Studies Hybridoma Bank), and anti-GFP (clone 3F8.2, Millipore) were used for immunoblotting analysis. Alexa Fluor 488 or 633-conjugated secondary antibodies were obtained from Invitrogen. The membranes were imaged for fluorescence using the Typhoon Trio plus imager (GE Healthcare Life Sciences, Inc.).

### Plasmids

Full-length human dysferlin cDNA, obtained from a clone DKFZp686K20163Q (RZPD, German) and a clone FLJ00175 (Kazusa DNA Research Institute, Japan), was subcloned into pEGFP-C3 (CLONTECH Laboratories, Inc.). To make CFP-dysferlin (CFP-dysferlin) and YFP-dysferlin (YFP-dysferlin) plasmids, the EGFP fragment in the EGFP-dysferlin construct was replaced with CFP or YFP which were amplified from pYFP-N1 and pCFP-N1 (CLONTECH Laboratories, Inc.). Different C2 domain constructs were generated by PCR. The CFP- and YFP-tagged constructs were used for the FRET studies, whereas YFP-tagged constructs were used in the biochemical and FCS experiments.

### Cell Culture and Transfection

HEK293 cells (Stratagene, Catalog No. 240073) were cultured in DMEM media supplemented with 10% FBS under 5% CO_2_ at 37°C. Transient transfection of HEK293 cells was performed by using Fugene 6 reagent (Roche) as suggested by the manufacturer. After 48 hours post-transfection, the cells were trypsinized and re-plated onto poly-D-lysine (Sigma-Aldrich) coated 4-well glass chambers (Lab-Tek, Nunc). For FRET experiment, 0.1 µg/well each of the plasmids with CFP and 0.5 µg/well each of the plasmids with YFP were co-transfected into HEK293 cells on 12-well plate. For biochemical experiments, 6.0 µg each of the plasmids with YFP was transfected into HEK 293 cells on 10-cm culture dish. After 48 hours post-transfection with the plasmids, cells were harvested in cold lysis buffer containing (mM): 50 Tris-HCl, pH 7.5, 150 NaCl, 1 EDTA, 0.5% n-dodecyl beta-D-maltoside (DDM), 0.2 phenylmethanesulfonylfluoride, and 1.0 benzamidine. Cell lysates were centrifuged at 14,000 rpm for 45 minutes and supernatants were collected. The protein samples were separated and blotted as described above.

### Electroporation and isolation of FDB muscle fibers

Electroporation-mediated gene transfer into *flexor digitorum brevis* (FDB) muscle of mice was performed as described previously [35]. Briefly, C57BL/6 and/or dysferlin-null mice at 6 to 20 weeks of age were anesthetized with ketamine/xylazine (87.5 mg/kg and 12.5 mg/kg, respectively). The feet were subcutaneously injected with 10 µL of the hyaluronidase solution (2 mg/ml) under the footpads using Hamilton syringe. After one hour, 15 µg CFP-dysferlin and 15 µg YFP-dysferlin plasmids (in 20 µl total volume) or otherwise specified were injected subcutaeneously under the footpads. The mice were allowed to fully recover in their cages under observation. After five to seven days post-electroporation, the mice were sacrificed and FDB muscles were dissected out. To isolate individual FDB muscle fibers, the FDB muscles were placed in Tyrode buffer containing 0.2% collagenase type 1A (Sigma-Aldrich) at 37°C for 15 minutes. After extensive wash with fresh Tyrode solution, the muscles were triturated with an open-mouth pipette to release individual FDB muscle fibers. The isolated FDB muscle fibers were plated onto 4-well glass chambers (Lab-Tek, Nunc), pre-coated with 1 µg/µl laminin (BD Biosciences), and imaged on fluorescence microscope or confocal microscope.

### Confocal and TIRF Microscopy

Cells transfected with fluorescent protein fusion constructs were imaged with a confocal microscope (TCS-SP5, Leica Microsystems), using the 514 nm line of an argon continuous laser as the excitation source. Fluorescence emission was collected with a 63×water immersion objective (HCX PL APO, 1.2 NA). Total internal reflection fluorescence (TIRF) imaging was also performed with an inverted fluorescence microscope as described [26]. To observe CFP fluorescence, a 449-nm diode laser was used for excitation. To observe YFP emission, a 514-nm argon laser was used for excitation. Fluorescence signals were collected through a back-thinned electron-multiplying charged couple device camera (Andor Technology) using the Metamorph software (Molecular Devices).

### Membrane repair assay

The membrane repair assay was performed essentially as previously described [1], [2], [44]. Briefly, isolated FDB muscle fibers were incubated in the Tyrode solution in 4-well glass-chamber (Lab-Tek, Nunc). The FDB muscle fibers were first imaged for CFP emission, and then 2.5 µM FM 1–43 (Invitrogen) was added into the chamber for the membrane repair assay. The cells with or without CFP-dysferlin fluorescence were selected. Membrane damage was induced in the presence of with a 2-photon confocal laser-scanning microscope (TCS-SP5, Leica Microsystems) coupled to a 10-W Argon/Ti:sapphire laser. After initial images were scanned, a 5-µm×5-µm area of the sarcolemma of the muscle fiber was injury with 1.5-s irradiation at full power. Fluorescence images were captured at 10-second intervals for 5 min after the damage. The fluorescence intensities at the damaged site were quantified by using the ImageJ software (NIH) and plotted against the time after the damage.

### FRET detection and analysis

FRET was quantified by acceptor-selective photobleaching and three-cube fluorescence microscopy.

#### Acceptor-selective photobleaching

Cells were imaged using inverted Nikon TE2000-U microscope equipped with a metal halide lamp, an APO 60×1.49 NA objective, and a back-thinned CCD camera (iXon 887; Andor Technology, Belfast, Northern Ireland). The CCD camera was cooled to −100°C, using a recirculating liquid coolant system (Koolance, Inc., Auburn, WA). CFP was excited at 427/10 nm and emission was collected with a 472/30 nm filter. YFP was excited at 504/12 nm and emission was collected with a 542/27 nm filter. CFP images were acquired for 100 ms and YFP for 40 ms, followed by 10-s exposure of YFP-selective photobleaching. Lamp output was set to 25% by a 0.6 ND filter in the light path. The intensity of this YFP photobleaching excitation was 230 micro-watts measured at the sample. At this power level, YFP fluorescence is reduced by more than 95% in 10 min, but CFP fluorescence is preserved. This protocol has been validated with standard samples [24] and used extensively [24], [25], [28], [32], [33]. Image acquisition was automated with Metamorph software. Fifty CFP-dysferlin and YFP-dysferlin images were collected and the entire procedure lasted about 6 minutes. Images were analyzed with NIH ImageJ software. Cell fluorescence intensities of CFP and YFP during photobleaching were measured and background signal was subtracted from each image. The pre- and post-bleach CFP fluorescence intensities were used to calculate FRET efficiency: FRET = 1−(F_prebleach_/F_postbleach_) [24], [25], [28], [32], [33]. To obtain the stoichiometry of the oligomeric complex that gives rise to FRET [24], [27], the CFP fluorescence emission was plotted against YFP fluorescence emission during photobleaching, and a linear regression was performed to examine the linearity of those two sets of signals. The maximal energy transfer efficiency (FRET_max_) and the dissociation constants (*K_D_*) were obtained from a hyperbolic fit with the equation FRET(*x*) = (FRET_max_)*x*/(*K_D_* + *x*), where *x* is the protein concentration [24], [25], [28], [32], [33]. In cell-based FRET assays the units are arbitrary because the protein concentration is determined from the fluorescence intensity [24], [25], [28], [32], [33]. YFP fluorescence intensity was used as an index of protein concentration.

#### Three-cube fluorescence microscopy

FRET was also measured with a “three-cube” method (E-FRET) [28], [31]. FRET was calculated according to: 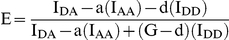 where I_DD_ is the intensity of fluorescence emission detected in the donor channel (472/30 nm) with 427/10 nm excitation; I_AA_ is the intensity of fluorescence emission detected in the acceptor channel with 542/27 nm emission and 504/12 nm excitation; I_DA_ is the intensity of fluorescence emission detected in the “FRET” channel with 542/27 nm emission and 427/10 nm excitation; a and d are cross-talk coefficients determined from acceptor-only or donor-only samples, respectively. We determined the a value of 0.074 for YFP and the d value of 0.7 for CFP. G is the ratio of the sensitized emission to the corresponding amount of donor recovery, which was 3.2 for this setup. The pixel-by-pixel FRET calculation process was automated using a Visual Basic for Applications macro in Excel.

### Fluorescence correlation spectroscopy and photon counting histogram analyses

For independent or uncorrelated processes, the contributions to the fluctuation of fluorescence signals of a given molecule in FCS studies are additive in nature [45]. A theoretical model for analyzing the FCS curves for our YFP labeled proteins includes the contribution from translational diffusion and the contributions from the on-off dynamics of YFP caused by single/triplet and protonated/unprotonated bright and dark states [46]. Such a model for a single species undergoing diffusion in a 3-dimensional Gaussian excitation volume is given by
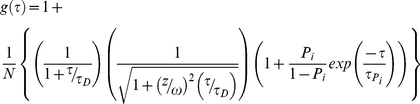
(1)where *N* denotes the average number of fluorescent molecules in the excitation volume defined by dimensions *z* and ω. *τ_D_* is the translation diffusion time of the protein and *P_i_* and *τ_Pi_* define the on-off dynamics of YFP. For the case of a mixture of molecules with different translation diffusion times, the equation 1 was modified as
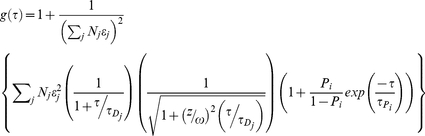
(2)where *N_j_* gives the average number of *j^th^* species in the excitation volume with a corresponding brightness of *ε_j_*. The measured FCS curves were fit to the corresponding theoretical model using a nonlinear fitting routine minimizing *χ^2^* and the correctness of the fits was judged by evaluating the residuals.

The diffusion coefficients were calculated from the measured *τ_D_* values according to 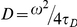. The diffusion coefficient for YFP was taken as a reference [47] for calculation of the diffusion coefficient for dysferlin. Assuming a spherical shape for the diffusing particle, the hydrodynamic radius was calculated for dysferlin using
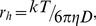
(3)where *k* is the Boltzmann constant (1.38×10^−23^ J/K), *T* the temperature and *η* the viscosity of the medium.

The PCH were analyzed using a nonlinear fitting routine with the molecular brightness (*ε_j_*) and average number of molecules in excitation volume (*N_j_*) as variables. For a mixture of 2 independent species of particles diffusing through the excitation volume, the PCH function is a convolution between the PCH functions of the individual species [37] yielding the average number of particles in the excitation volume (*N_1_* and *N_2_*) and the molecular brightness (*ε_1_* and *ε_2_*) for the first and second species. The molecular brightness and average particle number corresponding to the background autofluorescence was determined from the detergent solutions. A global analysis of multiple measurements was performed for a more robust determination of the fitted parameters.

For FCS and PCH studies, the YFP labeled protein samples were added into an 8-well glass chamber (Lab-Tek, Nunc) placed on the stage of a confocal microscope (TCS-SP5, Leica Microsystems). The samples were excited at 512 nm wavelength with an Ar-ion continuous wave laser that was attenuated to prevent photobleaching (the laser power was 4.5 µW at the sample). Fluorescence emission was collected with a 63× water immersion objective (HCX PL APO, 1.2 NA) and band pass filter (535–585 nm). Detection was by an avalanche photodiode (SPCM-AQRH, Perkin Elmer) and the time information of the detected photons was recorded by a photon counting card (ISS Inc.). FCS curves and PCH were generated by ISS-Vista software. The PCH were analyzed using a time bin width of 20 µs, which was much shorter than the translational diffusion time of the molecule in solution. Fluorescence traces were integrated for 100 seconds for each measurement. A 1-photon 3-dimensional Gaussian model was assumed for the excitation volume in our experiments.

### Statistical analysis

Data were presented as mean ± SEM, and statistical significance (p<0.05) was determined by the Student's t-test or one-way ANOVA using Prism 5.02 (Graphpad).
